# Multifaceted roles of superoxide dismutases (SODs) in cellular homeostasis and cancer progression: Redox regulation and therapeutic implications

**DOI:** 10.5937/jomb0-59010

**Published:** 2026-01-06

**Authors:** Duygu Aydemir, Nuriye Nuray Ulusu

**Affiliations:** 1 Istanbul University, Institute of Child Health, Department of Pediatric Basic Sciences, Istanbul, Türkiye; 2 Koç University, School of Medicine, Department of Medical Biochemistry, Sariyer, Istanbul, Türkiye; 3 Koç University Research Center for Translational Medicine (KUTTAM), Istanbul, Türkiye

**Keywords:** superoxide dismutases, ROS, cellular homeostasis, cancer progression, redox regulation, antioxidant systems, tumour microenvironment, oxidative stress, SOD1, SOD2, SOD3, superoksid-dismutaze, ROS, ćelijska homeostaza, progresija kancera, redoks regulacija, antioksidativni sistemi, tumorsko mikrookruženje, oksidativni stres, SOD1, SOD2, SOD3

## Abstract

Superoxide dismutases (SODs) are critical metalloenzymes that regulate cellular redox homeostasis by catalysing the dismutation of superoxide radicals into hydrogen peroxide and oxygen, thereby mitigating oxidative stress. Comprising three isoforms - SOD1 (Cu/Zn-SOD), SOD2 (Mn-SOD), and SOD3 (ecSOD) - these enzymes are localised in distinct cellular compartments, including the cytosol, mitochondria, and extracellular matrix, respectively. SODs play pivotal roles in cellular signalling, metabolism, and protection against reactive oxygen species (ROS)-mediated damage. Dysregulation of SOD expression and activity is implicated in various pathological conditions, particularly cancer, where they influence tumour initiation, progression, metastasis, and therapy resistance. Elevated SOD1 and SOD2 levels often promote oncogenic signalling and tumour survival, whereas SOD3 exhibits context-dependent roles, balancing tumour suppression and progression. Additionally, SOD mimetics, notably manganese-based compounds such as Mn-porphyrins and Mn-salens, have emerged as promising therapeutic agents that selectively modulate oxidative stress in cancer cells, thereby enhancing the efficacy of chemotherapy and radiotherapy while protecting normal tissues. This review explores the multifaceted roles of SODs in cellular homeostasis, their involvement in cancer pathogenesis, and the therapeutic potential of SOD mimetics in redox-based cancer strategies.

## Abstract (sr)

Superoksid-dismutaze (SOD) predstavljaju ključne metaloenzime u održavanju ćelijske redoks homeostaze, katalizujući dismutaciju superoksidnih radikala u vodonik-peroksid i kiseonik, čime se ublažava oksidativni stres. Tri izoforme ovih enzima - SOD1 (Cu/Zn-SOD), SOD2 (Mn-SOD) i SOD3 (ecSOD) - lokalizovane su u različitim ćelijskim odeljcima, uključujući citosol, mitohondrije i vanćelijski matriks. SOD enzimi imaju sustinsku ulogu u ćelijskoj signalizaciji, metabolizmu i zaštiti od oštećenja izazvanih reaktivnim kiseoničnim vrstama (ROS). Poremećaji u ekspresiji i aktivnosti SOD enzima su povezani sa brojnim patološkim stanjima, naročito sa kancerom, gde doprinose inicijaciji tumora, njegovoj progresiji, metastaziranju i razvoju rezistencije na terapiju. Povišena ekspresija SOD1 i SOD2 najčešće podstiče onkogenu signalizaciju i održavanje tumorskih ćelija, dok SOD3 pokazuje kontekstualno zavisne efekte, balansirajući između tumorske supresije i progresije. Dodatno, SOD mimetici, naročito jedinjenja na bazi mangana kao što su Mn-porfirini i Mn-saleni, izdvajaju se kao perspektivni terapeutski agensi, jer selektivno modulišu oksidativni stres u ćelijama raka. Na taj način povećavaju efikasnost hemioterapije i radioterapije, uz istovremenu zaštitu normalnih tkiva. Ovaj rad razmatra visestruke funkcije SOD enzima u ćelijskoj homeostazi, njihovu ulogu u patogenezi kancera i terapijski potencijal SOD mimetika u okviru redoks-zasnovanih strategija lečenja.

## Introduction

Superoxide dismutase (SOD, EC 1.15.1.1) enzymes are a group of metalloenzymes found in all eukaryotic and prokaryotic organisms that liven oxygen [1] [2]. SOD enzymes can detoxify free radicals and play a pivotal role in managing oxidative stress [3]. These enzymes primarily protect aerobic organisms from the toxic effects of reactive oxygen species (ROS). The mitochondria, essential for adenosine triphosphate (ATP) production, are the primary source of superoxide anions [4]. SOD enzymes can catalyse the conversion of superoxide anion (O_2_^•^^-^) into less harmful compounds, such as oxygen and hydrogen peroxide (H_2_O_2_), through a cyclic oxidation-reduction mechanism [5]. There are various ways to produce H_2_O_2_, but SOD enzymes mainly control H_2_O_2_ concentration [6]. For instance, plants, bacteria, fungi, and most other organisms except primates have urate oxidase enzymes that catalyse the production of H_2_O_2_ [7]. The best-known property of SOD enzymes is the antioxidant defence against oxidative stress via the scavenging of ROS [8]. SOD enzymes regulate essential processes in cellular signalling and primary cell metabolism, lipid metabolism, and inflammation involved in cell growth, proliferation, differentiation, cell survival, and apoptosis [9] [10], lipid peroxidation, oxidation of low-density lipoprotein in macrophages, lipid droplet formation, and adhesion of inflammation [11], and mitochondrial dysfunction [12]. SOD enzymes protect the cells from radical attack; therefore, they are accepted as a therapeutic agent against ROS-mediated diseases [13].

## Discussion

SOD enzymes are recognised as the primary enzyme in the detoxification/scavenging of O_2_^•^^-^, which is generated during cellular metabolism, environmental stresses, and programmed cell death [14] [15] [16]. SOD enzymes may trigger endogenous antioxidant enzymes for the neutralisation of ROS molecule H_2_O_2_ in a variety of pathological health conditions, for instance, xenobiotic metabolism, metabolic diseases, atherosclerosis, hypertension, angiogenesis, diabetes, cancer, pulmonary hypertension, nephropathy, edema, brain disease (Table 1) [12], cardiovascular diseases, cancer, respiratory diseases, skin diseases, renal, ocular diseases, neurological diseases, gastrointestinal diseases [17] [18] [19] [20] [21] [22]. It has been investigated that SOD enzyme activity is significantly decreased in patients with androgenetic alopecia [23] and those with Fanconi's anaemia [24]. SOD enzyme is a potential marker in patients with viral hepatitis E-induced liver failure. SOD enzyme level is increasing in patients with viral hepatitis E due to elevated oxidative stress during the pathogenesis of the disease [25].

**Table 1:** Classes, representative compounds, mechanistic roles, and therapeutic implications of superoxide dismutase (SOD) mimetics.

Class of SOD<br>Mimetics	Representative<br>Compounds	Mechanistic Role	Therapeutic Implications
Mn Porphyrins	MnTnHex-2-PyP^5+^,<br>MnBuOE-2-PyP^5+^,<br>HSJ-0017	Catalyse dismutation of superoxide and peroxynitrite; promote intracellular ROS accumulation in tumour cells; interfere with DNA repair; exert dual radiosensitising and radioprotective effects	Enhance efficacy of radiotherapy and<br>chemotherapy (breast, lung, melanoma,<br>glioblastoma); reduce normal tissue<br>toxicity; potential combinatorial use with<br>platinum-based drugs
Mn Salens	EUK-134,<br>EUK-189	Mimic both SOD and catalase<br>activity; attenuate oxidative damage;<br>induce cell cycle arrest and inhibit<br>migration	Suppress tumour proliferation,<br>particularly in breast cancer;<br>potential to overcome drug<br>resistance via redox modulation
Mitochondria-Targeted<br>Quinones	MitoQ10	Localise to mitochondria via<br>lipophilic cation; modulate<br>mitochondrial ROS and trigger<br>apoptosis and autophagy	Induce mitochondrial destabilisation and<br>tumour cell death; synergistic effects with<br>natural compounds (e.g., curcumin) in<br>redox-sensitive cancers
Nitroxides	Mito-TEMPO	Act as mitochondria-specific SOD<br>mimetics; scavenge mitochondrial<br>superoxide; inhibit NLRP3<br>inflammasome	Modulate inflammatory signalling and<br>tumour microenvironment; protective role<br>in ischemia-reperfusion injury and<br>inflammation-associated malignancies
Mangafodipir<br>Derivatives	Mangafodipir,<br>Calmangafodipir	Initially developed as MRI contrast<br>agents; exhibit MnSOD-mimetic<br>and iron-chelating activity;<br>Calmangafodipir is engineered to<br>reduce Mn toxicity	Enhance chemotherapy efficacy<br>(oxaliplatin, platinum derivatives); protect<br>against haematological toxicity and<br>chemotherapy-induced peripheral<br>neuropathy

SOD enzymes are localised in the cytosol, mitochondria, chloroplasts [26], nucleus, lysosomes, peroxisomes, extracellular matrix, cell surface, and extra-cellular fluids (Table 1) [12]. They require metals for catalytic activity and can be classified according to the type of metal cofactors they use. For instance, copper-zinc superoxide dismutase, also known as SOD1, is commonly found in eukaryotes [27], while the iron-manganese-containing superoxide dismutase enzyme is found in bacteria and chloroplasts [28]. Ni-containing SOD enzymes are found in prokaryotes such as Streptomyces sp. [29]. On the other hand, three SOD enzymes are found in plants with different metal cofactors: Fe-SOD, Cu-SOD, and Mn-SOD [30]. Alternatively, SOD isoenzymes are named SOD1, SOD2, and SOD3, and their cellular localisation is also essential. SOD1 or Cu, Zn-SOD enzyme is generally accepted as the central enzyme [31]. Found in the cytosol and mitochondrial intermembrane space (IMS), nucleus, and endoplasmic reticulum [32], lysosomes, and peroxisomes [12]. Mn-SOD is SOD2, found in the mitochondrial matrix and mitochondrial inner membrane [33], whereas the one found in the extracellular matrix is named SOD3 [33] [34].

**Figure 1:**
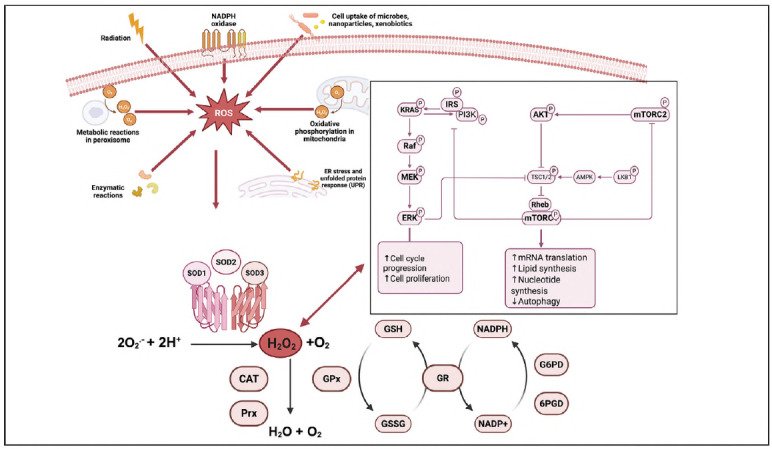
The role of Superoxide Dismutase (SOD) enzyme in the cell is associated with signalling pathways involved in cancer progression.<br>This figure illustrates the potential involvement of SOD enzymes in cancerous cells. SOD enzymes, which play a crucial role in metabolising reactive oxygen species (ROS), are present in both normal and cancer cells. In normal cells, SOD enzymes help maintain cellular redox balance by converting superoxide radicals (O_2_^-^) into less reactive molecules, such as hydrogen peroxide (H_2_O_2_) and oxygen. In contrast, in cancer cells, altered expression or activity of SOD enzymes may lead to an imbalance in ROS levels, contributing to genomic instability, tumorigenesis, and metastasis.

SOD enzymes convert two O_2_^•^^-^ into H_2_O_2_, which is further catalysed into H_2_O and O_2_ by catalase (CAT) and glutathione peroxidase (GPx) enzymes. GPx and GST enzymes are antioxidant enzymes that require GSH to function, and an increased GSSG/GSH ratio is the cell's primary biomarker for oxidative stress status. Altered function in the antioxidant defence system causes enhanced accumulation of ROS associated with various disease pathogeneses [35]. Oxidative stress is described by the increased accumulation of ROS and decreased antioxidant defence in the cell. Decreased SOD levels lead to the accumulation of ROS, primarily O_2_^•^^-^, within the cell, triggering nucleophilic reactions. Since O_2_^•^^-^ deprotonates phenols, alcohols, and thiols and hydrolyses esters, it phosphorylates various types of proteins, including PKC, PKD (protein kinase D), PKB (Akt) (protein kinase B), and mitogen-activated (MAPK) kinases, p42/44, p38, ERK, and PI3K [36]. Excessive ROS, including O_2_^•^^-^, attacks DNA, lipids, and proteins, causing DNA damage and lipid peroxidation [18]. Most types of cancer elevate their intrinsic ROS levels to promote oncogenic pathways, including genetic alterations, metabolic reprogramming, and metastasis. Thus, regulating oxidative stress metabolism in the tumour microenvironment and peripheral tissues is vital for controlling oncogenesis and developing hemotherapeutic approaches [37]. Since the SOD family is the central regulator of oxidative stress metabolism and antioxidant response, recent studies have highlighted the significant role of the SOD family in cancer progression (Figure 1) [38].

### The role of the SOD enzymes in cancer pathogenesis

### SOD1

SOD1 enzyme is a 32 kDa homodimeric metalloprotein containing Cu and Zn, and provides 80% of all SOD activity in the cell. SOD1 enzyme was discovered 50 years ago and is associated with the pathogenesis of various diseases, including cancer, neurological disorders, reduced fertility, ageing, metabolic disorders, and muscle wasting [39]. SOD 1 enzyme transcriptionally regulated, and this isoenzyme's activity is also controlled by posttranscriptional modifications (PTM). PTM can regulate conformation, function, and localisation [40]. SOD1 has a controversial role in cancer progression because decreased SOD levels lead to an increase in ROS levels associated with oncogenesis. On the other hand, cancer cells require antioxidant enzymes, such as the SOD family, to prevent oxidative stress-induced cell damage and apoptosis [41]. Increased SOD1 activity results in the H_2_O_2_ accumulation in the cell that triggers various signaling cascades, including AMP-activated protein kinase (AMPK), mitogen-activated protein kinase (MAPK), nuclear factor-кВ (NF-kB), c-Jun N-terminal kinase (JNK), phosphatidylinositol 3-kinase-Akt (PI3K-Akt), Janus kinase-signal transducers and activator of transcription (JAK-STAT), activator protein-1 (AP-1), nuclear factor (erythroid-derived 2)- related factor 2 (Nrf2), hypoxia-inducible factor (HIF), Ras, and Rac involved in the progression, tumorigenesis, angiogenesis, and metastasis of cancer cells [42].

SOD1 plays a regulatory role in H_2_O_2_-mediated signalling, and the overexpression of SOD1 activates the MAPK, JNK, and Akt pathways, as well as the AP-1, TNF-α, and JAK-STAT pathways involved in torigenesis [43]. On the other hand, SOD1 localised in the nucleus regulates transcription of the stress response genes under enhanced oxidative stress in the cell. For instance, SOD1 induces glycolysis and suppresses oxidative phosphorylation [44]. Overexpression of SOD1 has been reported in various types of cancers, such as non-small cell lung cancer (NSCLC), pancreatic cancer, liver cancer, brain cancer, nasopharyngeal carcinoma (NPC), leukaemia, and breast cancer [45]. Knockdown or pharmaceutical inhibition of SOD1 blocks the proliferation of NSCLC, fibrosarcoma, pancreatic cancer, and breast cancer cells [46]. SOD1 overexpression has been linked to cisplatin resistance in human ovarian cancer cells, and targeting SOD1 results in hemosensitivity in these cell lines [47].

### SOD2

Human SOD2 (MnSOD) is a nuclear-encoded enzyme located in the mitochondria and controlled at transcriptional, translational, and post-translational levels. The SOD2 gene encodes an 88-kDa homotetrameric protein, and the enzyme's active site contains manganese [48]. Nuclear factor kappa-light-chain-enhancer of activated В cells (NF-kB), specificity protein 1 (Sp1), activating protein-1 (AP-1), p53, and CCAAT binding protein (C/EBP) directly regulate SOD2 transcription under oxidative stress conditions via directly binding its promoter. On the other hand, pro-inflammatory cytokines (TNF-α, IL-1β, IFN-γ), growth factors (TGF-β), fibroblast and epidermal growth factor, platelet-derived growth factor, endotoxins, extracellular factors (lipopolysaccharide, c-AMP UV), nitric oxide, ionising radiation, viral infection, hypoxia, bacterial infection, and anticancer drugs (tamoxifen, paclitaxel, vinblastine, vincristine) can stimulate SOD2 transcription [38] [48] [49] [50] [51] [52].

Mitochondria are the primary source of oxidative stress in the cell due to oxidative phosphorylation; thus, SOD2 plays a vital role in antioxidant defence in both healthy and cancer cells. Homozygous knockout of SOD2 causes lethality in mice; however, homozygous knockout models of SOD1 and SOD3 have reportedly survived, according to the literature [53]. SOD2 overexpression enhances mitochondrial function by scavenging O_2_^•^^-^ produced with the mitochondria [54]. The role of SOD2 in cancer pathogenesis is complex, as changes in SOD2 expression and activity are tumour-type dependent. Decreased SOD2 expression is frequently observed during tumour initiation; however, SOD2 levels increase during tumour progression and metastasis [55]. Although increased SOD2 levels enable cells to cope with oxidative stress by scavenging O_2_^•^^-^ an SOD2-dependent increase in H_2_O_2_ leads to a shift in the cell's metabolism towards a H_2_O_2_-dependent redox signalling status [56].

Increased SOD2 levels are associated with a 10-fold increase in the risk for pancreatic cancer; elevated H_2_O_2_ levels induced by SOD2 overexpression also led to increased GPx activity [57]. Enhanced SOD2 induces tumorigenic and proangiogenic pathways such as VEGF, AKT, and HIF1α in thyroid, pancreatic, colon, and breast cancers [58] [59]. Since increased SOD2/H_2_O_2_, SOD2/CAT, and SOD2/GPx ratios are associated with tumour progression and metastasis in prostate, lung, and colon cancers, these ratios are considered valuable predictive biomarkers for the aforementioned cancer types [60]. High SOD2 expression was linked to lymph node metastasis in oral squamous cell carcinoma (OSCC) [61].

SOD2 is downregulated in follicular thyroid cancer, and decreased expression of SOD2 is directly correlated with poor survival of patients having aggressive thyroid or adrenal cancer [62]. The rs4880 polymorphism in exon 2 of SOD2 at position 16 is characterised by changing alanine (Ala) to valine (Val). This polymorphism leads to decreased mRNA expression and stability in SOD2, resulting in alterations in the enzyme's import into the mitochondria found in lung and oesophageal cancers [63]. Reduced SOD2 transcription correlates with increased mortality in hepatocellular carcinoma patients harbouring p53 mutation, indicating a tight relationship between p53 and SOD2 [64].

### SOD3

SOD3 is the only antioxidant enzyme present in the extracellular matrix of restricted tissue and cell types; however, this enzyme has also been found in the nucleus and is trafficked via the endo-lysosomal system within the cell [65]. The role of SOD3 enzymes in cancer development is multifaceted; they are involved in redox signalling, function as second messengers in regulatory pathways, contribute to maintaining genomic stability, and play a protective role in preventing carcinogenesis [66]. The extracellular SOD (ecSOD; SOD3) has unique functions in cellular transduction, oxidative tumour microenvironment, tumour growth, metastasis, and recurrence [65]. SOD3 is highly expressed in the lung, placenta, and cardiac endothelium, whereas it is moderately expressed in the pancreas, kidney, uterus, cartilage, brain, eye, skeletal muscle, and adipose tissue [67]. After SOD3 is synthesised, it is bound to the cell surface proteoglycans via its positively charged heparinbinding domain (HBD). The HBD domain is cleaved by proteases, allowing SOD3 to be distributed into the extracellular milieu and the circulatory system [68]. The primary function of the SOD3 is to control the radical levels in the cell via catalysing O_2_^•^^-^ into H_2_O_2_, inhibiting the production of the ^•^OH via the Fenton and Haber Weiss reaction, and preventing the O_2_^•^^-^ -mediated oxidation of NO^•^ [65].

NO^•^ is produced by nitric oxide synthases (NOSs) and regulates the vessel relaxation in endothelial cells, neurotransmitter function, and macrophage and neutrophil functions. Firstly, the tissue-protective effects of SOD3 on cardiac tissue have been reported, and SOD3 administration has been shown to reduce cardiovascular damage, as documented in the literature [69]. O_2_^•^^- ^mediated peroxynitrite (ONOO^-^) production from NO^•^ has adverse effects on cellular signalling; on the other hand, NO^•^ exerts both cancer-promoting and anticancer effects. ONOO attacks lipids, DNA, and proteins via radical-mediated reactions that trigger cell death mechanisms. Enhanced ONOO^-^ levels have been observed in stroke, cardiac arrest, myocardial infarction, diabetes, chronic shock, inflammatory diseases, neurodegenerative disorders, and cancer; thus, NO^•^/ONOO^-^ levels have a significant role in cancer pathogenesis [70].

Decreased SOD3 transcription and translation have been observed in patients with pancreatic cancer. This decrease in SOD3 is associated with a corresponding reduction in mean survival from 11.0 to 6.5 months in patients with pancreatic adenocarcinoma [71]. Upregulation of SOD3 leads to the inhibition of cell proliferation, clonogenic capacity, and invasion in a dose-dependent manner via decreasing VEGF and HIF-1α protein levels. Since VEGF and HIF-1α induce angiogenesis, SOD3 inhibits the blood flow into the tumour. On the other hand, SOD1 and SOD3 upregulation via Mirk/Dyrk1B kinase maintains cell growth by reducing ROS levels in the quiescent pancreatic cell lines Panc1 and SU86.86 in vitro [72]. SOD3 upregulation decreased cell proliferation in melanoma cells via decreased IFNγ and VEGF levels [73]. SOD3-induced pathways in tumours connect vascular normalisation and T-cell diapedesis. Perivascular SOD3 prevents the oxidation of nitric oxide (NO), leading to increased endothelial cell (EC) NO levels, which inhibit prolyl hydroxylase domain (PHD) activity and cause the nuclear accumulation of HIF-2α. HIF-2α then upregulates vascular endothelial cadherin (VEC) and specific WNT ligands, which reduce vascular permeability and contribute to vascular normalisation [74]. The upregulation of SOD3 reduces tumour growth and liver metastasis in colorectal cancer, indicating its potential as a diagnostic and prognostic marker in the treatment of colorectal cancer [75].

On the other hand, according to metabolic and proteomic studies, melanoma tumours recovered from chemotherapy showed enhanced SOD3 activity [76]. SOD3 has protective effects on normal lung function, and downregulated SOD3 has been reported in lung cancer between stages I-IV Overexpression of SOD3 reduced invasion and clonogenic survival by inhibiting NF- B in lung cancer [77].

### SOD mimetics in diseases

Superoxide dismutase (SOD) mimetics are synthetic or low-molecular-weight compounds designed to replicate the activity of native SOD enzymes, which catalyse the dismutation of superoxide radicals into oxygen and hydrogen peroxide. These mimetics, particularly those based on manganese (Mn), have garnered significant interest due to their potential in mitigating oxidative stress-related pathologies, including cancer, neurodegenerative diseases, and inflammatory disorders. Mn-based SOD mimetics, including Mn-porphyrins, Mn-salen complexes, and Mn-pyridyl ligands, emphasise their structural versatility, catalytic mechanisms, and biological efficacy. Preclinical studies suggest these mimetics offer therapeutic advantages by modulating redox signalling and protecting tissues from oxidative damage, with some advancing toward clinical evaluation in various diseases, including head and neck, breast, brain, skin, and lymphoma cancers. On the other hand, Mn-based SOD mimetics enhance the efficacy of chemotherapy and radiotherapy, while also reducing tumour growth [78].

### MnSOD mimetics in cancer therapy: A focus on compound classes and mechanistic insights

MnSOD mimetics represent a promising class of therapeutic agents in oncology due to their ability to selectively modulate redox homeostasis in tumour cells versus normal cells. Several subclasses of these mimetics, particularly Mn-based complexes and mitochondrial-targeted antioxidants, have demonstrated significant efficacy in preclinical cancer models. This section outlines the current understanding of the anticancer mechanisms and therapeutic potential of major MnSOD mimetics (Table II) [79]. Table 2

**Table 2:** Isoenzyme-specific localisation, physiological roles, pathological involvement, and therapeutic implications of superoxide dismutases (SODs). SOD1, SOD2, and SOD3 are critical antioxidant enzymes that regulate cellular and extracellular redox balance by catalysing the dismutation of superoxide radicals into hydrogen peroxide and oxygen.

Isoenzyme	Cellular<br>Localisation	Physiological Role	Role in Cancer and Pathology	Therapeutic Implications
SOD1<br>(Cu/Zn-SOD)	Cytosol,<br>mitochondrial<br>intermembrane<br>space, nucleus,<br>ER,<br>peroxisomes,<br>lysosomes	Detoxifies cytosolic<br>superoxide radicals;<br>regulates redox-sensitive<br>signalling; balances<br>glycolysis vs. oxidative<br>phosphorylation	Overexpressed in lung,<br>pancreatic, liver, breast<br>cancers, leukaemia, and NPC;<br>promotes tumour growth and<br>survival via MAPK, NF-kB,<br>PI3K/Akt, JAK-STAT, HIF<br>pathways; contributes to<br>chemoresistance (cisplatin)	Targeting SOD1 sensitises<br>tumours to chemotherapy;<br>potential biomarker for<br>therapy resistance; SOD1<br>inhibitors may block<br>oncogenic signalling
SOD2<br>(Mn-SOD)	Mitochondrial<br>matrix and inner<br>membrane	Primary mitochondrial<br>antioxidant defence;<br>essential for survival;<br>regulates oxidative<br>phosphorylation	Tumour-type dependent:<br>reduced in initiation,<br>elevated in progression/<br>metastasis; high SOD2/H2O2<br>promotes angiogenesis (VEGF,<br>HIF-1α, AKT); linked to<br>prostate, thyroid,<br>pancreatic, and colon<br>cancer progression	Inhibition enhances<br>sensitivity to chemotherapy<br>(e.g., 5-FU in gastric<br>cancer); SOD2 expression<br>ratios (SOD2/GPx,<br>SOD2/cAT) serve as<br>prognostic biomarkers;<br>SOD2 polymorphisms<br>linked to cancer risk
SOD3<br>(Ec-SOD)	Extracellular<br>matrix, circulation,<br>nucleus (via endolysosomal<br>trafficking)	Regulates extracellular<br>ROS; preserves NO<br>bioavailability; maintains<br>vascular and tissue<br>integrity	Downregulated in<br>pancreatic and lung cancer (correlates<br>with poor<br>survival); overexpression suppresses<br>invasion and angiogenesis;<br>context-dependent tumour<br>effects	Potential diagnostic/<br>prognostic biomarker<br>(lung, pancreatic, colorectal<br>cancer); therapeutic<br>upregulation may inhibit<br>angiogenesis and metastasis;<br>protective in normal tissues<br>against oxidative injury

### Mn porphyrins

Among the MnSOD mimetics, manganese porphyrins are the most extensively studied, particularly for their potential to enhance the efficacy of radiotherapy and chemotherapy. MnTnHex-2-PyP^5+^ (MnTnHex) is a highly lipophilic Mn porphyrin that exhibits potent radio-sensitising effects in tumour cells. It improves the therapeutic efficacy of ionising radiation in breast cancer and melanoma models by promoting intracellular reactive oxygen species (ROS) accumulation and impairing DNA repair mechanisms. Additionally, MnTnHex has demonstrated synergism with cisplatin in non-small cell lung carcinoma (NSCLC) and renal carcinoma models, where it amplifies cytotoxicity and inhibits cancer cell migration, suggesting potential in combination chemotherapy regimens [78].

*MnBuOE-2-PyP (MnBuOE)* similarly exerts dual pro-oxidative and protective effects. In glioblastoma, ovarian, and lung cancer models, MnBuOE selectively increases oxidative stress in tumour cells, promoting apoptosis when used in conjunction with chemotherapeutic agents such as carboplatin, cisplatin, and tumour necrosis factor-related apoptosis-inducing ligand (TRAIL). MnBuOE has shown radioprotective effects in normal tissues, notably preserving hippocampal neurogenesis during cranial irradiation. This selective action enhances the therapeutic window of cancer treatment protocols [79].

*HSJ-0017 *is a recent Mn porphyrin compound combining anti-inflammatory and antitumor properties. In sarcoma models, HSJ-0017 augments the efficacy of chemotherapy and radiotherapy while concurrently mitigating treatment-induced toxicity. Its dual function underscores its value as a cytotoxic enhancer and a tissue-protective agent [80].

### Mn salens

Mn salen complexes, such as EUK-134, mimic both SOD and catalase activity and have demonstrated efficacy in cancer settings, particularly breast cancer. EUK-134 reduces intracellular superoxide and hydrogen peroxide levels, induces cell cycle arrest at the G2-M phase, and inhibits cell migration and adhesion. These actions collectively contribute to suppressed tumour proliferation and may counteract mechanisms underlying drug resistance. EUK-134's redox-modulating effects position it as a promising adjunct to existing therapies targeting redox-sensitive tumour pathways [80].

### MitoQ10

Though not manganese-based, MitoQ10 functions as a mitochondria-targeted antioxidant with mechanistic parallels to MnSOD mimetics. It comprises a ubiquinone moiety linked to a triphenylphosphonium cation, facilitating mitochondrial accumulation. MitoQ10 induces mitochondrial destabilisation in cancer models, leading to apoptosis and autophagy. Its combination with agents such as curcumin further enhances tumoricidal activity, indicating potential utility in redox-sensitive cancer therapy [81].

### Nitroxides

Nitroxide derivatives, particularly Mito-TEMPO, offer mitochondria-specific modulation of oxidative stress without containing manganese. Mito-TEMPO scavenges mitochondrial ROS and inhibits inflammatory signalling pathways such as the NOD-like receptor pyrin domain-containing 3 (NLRP3) inflammasome. These properties are particularly relevant in malignancies characterised by mitochondrial dysfunction and inflammation, positioning Mito-TEMPO as a potential therapeutic adjunct to modulate the tumour microenvironment and redox signalling [82].

### Mangafodipir and Calmangafodipir

Initially developed as MRI contrast agents, Mangafodipir and its derivative Calmangafodipir have been repurposed for their MnSOD-mimetic properties. Calmangafodipir has been engineered to reduce manganese-associated toxicity through partial substitution with calcium. It has demonstrated the ability to enhance the efficacy of chemotherapy agents, such as oxaliplatin, while concurrently mitigating associated haematological toxicity and peripheral neuropathy. This dual action improves the therapeutic index of chemotherapeutic regimens and supports their development as chemoprotective adjuvants [83].

MnSOD mimetics, including Mn porphyrins, salens, mitochondria-targeted quinones, and related compounds, offer diverse and complementary mechanisms of action in cancer therapy. Their ability to selectively modulate oxidative stress within the tumour microenvironment presents a novel and multifaceted approach to enhancing therapeutic outcomes while minimising damage to normal tissues. Further clinical investigation is warranted to translate these promising preclinical results into effective adjuncts for standard cancer treatments [84].

## Conclusion

Superoxide dismutase (SOD) enzymes are central components of the cellular antioxidant defence system, critically involved in regulating redox balance by catalysing the dismutation of superoxide radicals into hydrogen peroxide. The three isoenzymes, SOD1, SOD2, and SOD3, each play distinct yet overlapping roles in maintaining cellular homeostasis and modulating oxidative stress in physiological and pathological contexts. Their dysregulation is closely associated with cancer initiation, progression, metastasis, and resistance to therapy. SOD1 and SOD2 are intimately linked to redox-sensitive oncogenic signalling pathways and contribute to tumour cell survival under oxidative stress. The SOD1-mTORC1 axis supports tumour adaptation to hypoxia and nutrient deprivation, while SOD2 expression correlates with poor prognosis and resistance to chemotherapeutic agents in several cancers. SOD3, although primarily protective in normal tissue, has context-dependent roles in tumour biology, influencing angiogenesis, invasion, and immune modulation.

Furthermore, the development of MnSOD mimetics, such as Mn porphyrins, salens, and mitochondria-targeted antioxidants, has opened new avenues in cancer therapy. These agents can selectively amplify oxidative stress in tumour cells while protecting normal tissues, enhancing the efficacy of chemotherapy and radiotherapy. Their dual action highlights the therapeutic potential of redox modulation in oncology. Overall, SOD enzymes not only serve as key modulators of oxidative stress but also emerge as critical biomarkers and therapeutic targets in cancer. Continued investigation into their regulatory mechanisms, interactions with cellular signalling networks, and pharmacological modulation through mimetics is essential for advancing redox-based strategies in precision cancer therapy.

## Dodatak

### Ethical approval

No ethical approval is required for this study.

### Authors contributions

DA and NNU were responsible for conceptualisation, writing the original manuscript, and revising it.

### Funding

No funding.

### Conflict of interest statement

All the authors declare that they have no conflict of interest in this work.
